# Perforated Gastric Cancer: A Case Report and Literature Review

**DOI:** 10.7759/cureus.51767

**Published:** 2024-01-06

**Authors:** Kai Wang, Aye Thida, Gyuhee Seong, Edwin Chiu

**Affiliations:** 1 Department of Internal Medicine, St. George's University School of Medicine, New York, USA; 2 Department of Hematology and Oncology, SUNY (State University of New York) Downstate Health Sciences University/Kings County Hospital Center, New York, USA; 3 Department of Medicine, SUNY (State University of New York) Downstate Health Sciences University/Kings County Hospital Center, New York, USA

**Keywords:** gastric perforation, laparoscopy, hyperthermic intraperitoneal chemotherapy, gastric cancer (gc), perforated gastric cancer

## Abstract

Gastric cancer perforation is a rare but life-threatening complication of gastric cancer. We present the case of a 53-year-old male with acquired immune deficiency syndrome (AIDS) who presented to the emergency department with severe abdominal pain, was found to have an acute abdomen, and was eventually diagnosed with gastric perforation due to metastatic gastric cancer. This case highlights the challenges in diagnosing and managing perforated gastric cancer and discusses the surgical management options, including the use of laparoscopic techniques and the role of chemotherapy, particularly hyperthermic intraperitoneal chemotherapy (HIPEC).

## Introduction

Gastric perforation is characterized by a rupture in the gastric wall, leading to the spillage of gastric alimentary contents into the peritoneal cavity, thus causing a surgical emergency with potentially life-threatening outcomes. Patients initially present with sudden severe, sharp abdominal pain in the epigastric area, nausea, vomiting, and diarrhea with tachycardia, hypotension, and fever [1]. If unrecognized and untreated, this can progress to septic shock. Laboratory results may demonstrate leukocytosis, metabolic acidosis, and hyperamylasemia [2].

Computed tomography (CT) of the abdomen has been established as the most valuable imaging technique for identifying the presence, location, and potential cause of gastrointestinal tract perforation. An upright chest X-ray characteristically shows free air under the diaphragm (pneumoperitoneum). A CT abdomen with contrast would show free air, mesenteric fat stranding, and bowel wall thickening, in addition to the location of the perforation [2].

Gastric perforation is most commonly caused by peptic ulcer disease (PUD). However, it may be secondary to trauma, nonsteroidal anti-inflammatory drugs (NSAIDs) use, ischemia, gastric volvulus, or, in rare cases, gastric cancer (GC), and chemotherapy [2].

We discuss a case of gastric perforation due to metastatic gastric cancer.

## Case presentation

**Figure 1:**
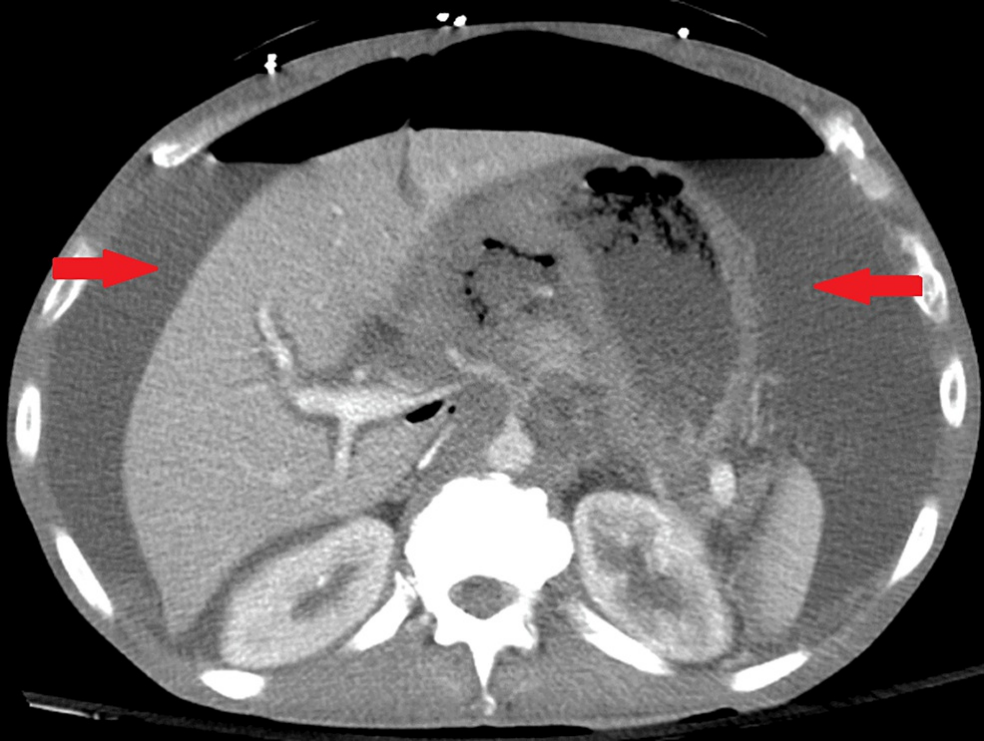
Computed tomography angiogram of the abdomen and pelvis showed large-volume pneumoperitoneum and ascites (arrow), suggestive of perforated viscus and peritonitis

A 53-year-old male with multiple comorbidities, including acquired immune deficiency syndrome (AIDS) (cluster of differentiation 4 (CD4) 14 cells/microL, viral load 109,294 copies/mL), presented with constant, generalized abdominal pain for five days, accompanied by subjective fever. He reported “black” vomit that became blood-streaked. On arrival at the ED, the patient was in acute distress. Vitals were significant for a pulse rate of 135/min, respiratory rate of 36/min, and blood pressure of 55/45 mmHg. He appeared cachectic and had a distended abdomen with diffuse tenderness and guarding, typical of an acute abdomen. Labs were notable for elevated lactate levels of 8.3 mmol/L and high anion gap (anion gap 21mEq/L) metabolic acidosis, suggesting sepsis. A CT angiogram of the abdomen and pelvis showed large-volume pneumoperitoneum and ascites, concerning perforated viscus and peritonitis secondary to gastric perforation (Figure 1). CT angiogram of the chest showed no metastasis.

The patient was then started on broad-spectrum antibiotics and vasopressors and was subsequently admitted to the surgical ICU for septic shock and pneumoperitoneum. The patient also received therapeutic paracentesis performed to relieve abdominal compartment syndrome.

The patient was intubated and underwent emergent exploratory laparotomy, which revealed a 3.5 cm perforation in the lesser gastric curvature along with diffuse carcinomatosis. Gastric and omental biopsies were taken. Due to the patient's hemodynamic instability, the decision was made to perform wide drainage of the gastric perforation. A large Malecot drain was placed into the perforation and secured through the right abdominal wall, along with a Blake drain on the posterior side and two Jackson-Pratt (JP) drains on the anterior and posterolateral sides to the Malecot drain. The abdomen was then left open for a planned second look/revision and washout due to peritonitis.

**Figure 2:**
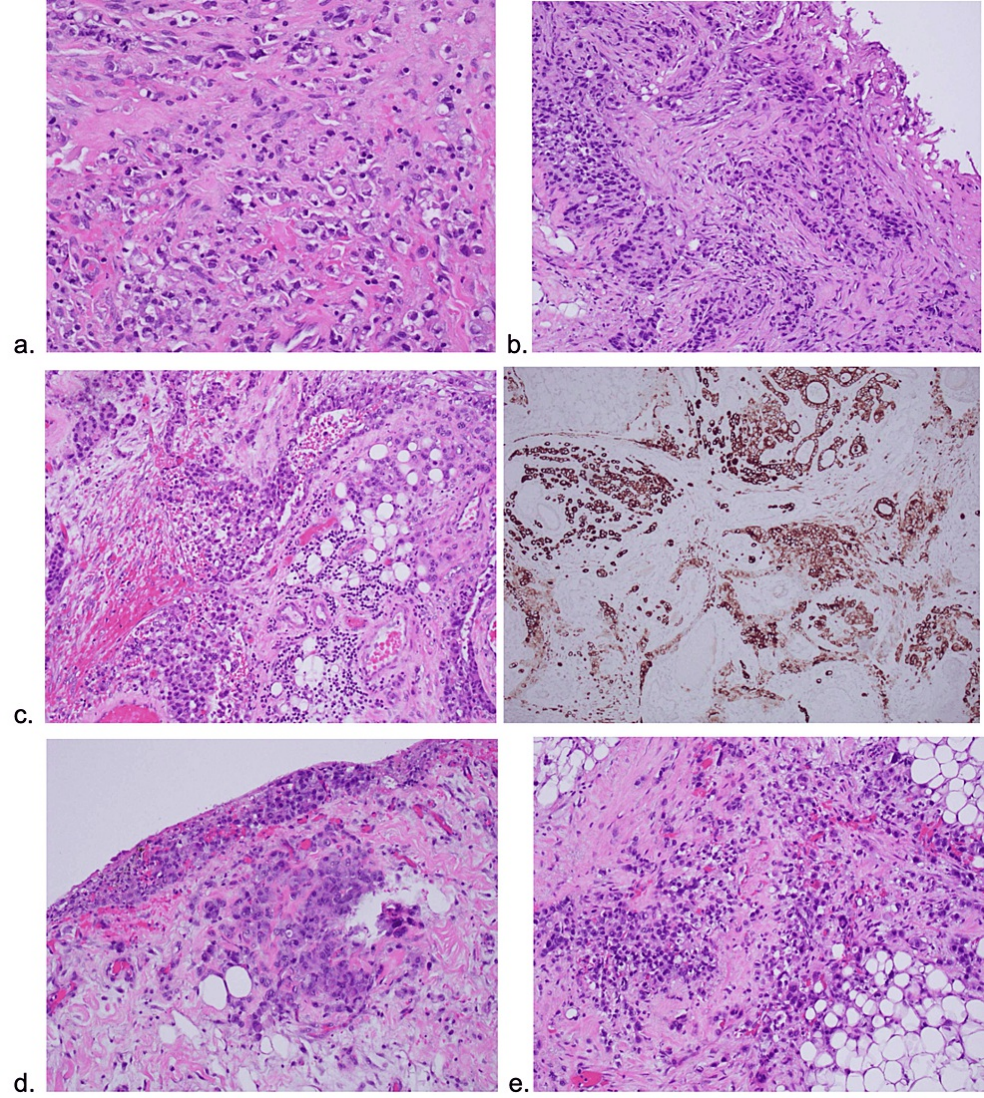
Histology of gastric ulcer biopsy a) Hematoxylin and eosin (H&E) of the gastric ulcer biopsy showing poorly differentiated adenocarcinoma. b) H&E of the peritoneal implant biopsy showing poorly differentiated adenocarcinoma. c) H&E of the omental biopsy showing poorly differentiated adenocarcinoma. Immunohistochemistry (IHC) showing CK7/CK20/CDX2 positive and MUC2/MKX3.1 negative. d) H&E of the peritoneum, mesenteric small bowel biopsy, and positive for poorly differentiated adenocarcinoma. e) H&E of the mesocolon soft tissue biopsy positive for poorly differentiated adenocarcinoma. All biopsies had histomorphology and immunostains supportive of metastatic adenocarcinoma of gastric origin.

The next day, the patient received a revision exploratory laparotomy with a peritoneal biopsy. The carcinomatosis involved the transverse colon, small bowel mesentery, and omentum. Frozen sections of peritoneal implants were positive for malignancy. The decision was made at that time to maintain the original wide drainage without any further intervention. The fascia, subcutaneous tissue, and preperitoneal muscles were closed while the skin was left open. Pathology of the omental biopsy showed poorly differentiated adenocarcinoma with immunohistochemistry stains positive for CK7, CK20, and CDX2, and negative for MUC2 and NKX3.1. Taken together, the adenocarcinoma was likely of gastric origin and staged as TxNxM1 stage IV gastric adenocarcinoma (Figure 2).

Following the surgery, the patient’s clinical status remained in critical condition and required continuous vasopressor and ventilator support. The case was reviewed by a multidisciplinary tumor board with a consensus on the best supportive care and hospice. The patient's healthcare proxy requested terminal extubation and comfort care. He passed away on hospital day 30.

## Discussion

Gastric cancer (GC)**

GC is the third leading cause of death from cancer worldwide [3]. GC is most commonly adenocarcinoma, but it can also be a gastrointestinal stromal tumor (GIST), primary gastric lymphoma, or gastric hepatoid adenocarcinoma. GC can also be the result of metastasis or an infection (Helicobacter pylori). Early diagnosis of GC is often delayed because patients tend to be asymptomatic in the early stages of the disease and would only be discovered when the patient presents with persistent abdominal pain and weight loss [4].

Currently, the gold standard of diagnosis of GC is with an esophagogastroduodenoscopy (EGD) with endoscopic biopsy [4]. Specific markers and histological features from the biopsy can aid in tumor localization and primary disease identification [4]. Once the diagnosis of GC is achieved with tissue biopsy, a complete evaluation should be done to determine the tumor, node, metastasis (TNM) staging using CT imaging [5]. An endoscopic ultrasound (EUS) is recommended to evaluate the gastric wall for depth of cancer invasion in localized cases [5]. The five-year survival rate of GC can reach up to 99% in cases of early-stage disease and decreases to less than 30% when diagnosis is delayed, leading to advanced disease [6]. In countries with a high incidence of GC, such as Korea and Japan, the nationwide implementation of early GC screening has decreased the mortality of GC by 30% [7]. According to the American Cancer Society, the incidence of gastric cancer is typically low in the United States (approximately 1.5% of all newly diagnosed cancers). However, it is almost double in Asians, African Americans, and Hispanics [8], making the idea of early GC screening with upper endoscopy possibly beneficial in these populations.

Perforation of gastric cancer

Perforated gastric cancer (PGC) is a rare condition that accounts for less than 1% of cases of acute abdomen and less than 5% when gastric cancer reaches an advanced stage [9]. It represents a significant oncologic emergency, second only to major bleeding. Despite its importance, the management of PGC remains controversial due to the absence of clinical guidelines supporting a specific algorithm in emergencies. In these cases, surgery serves a dual purpose: treating life-threatening peritonitis and aiming to cure gastric cancer.

The challenge of diagnosing PGC lies in its similarity of symptoms to other causes of gastric perforation such as PUD. This means that unless there is a preoperative diagnosis of gastric cancer, which is only present in approximately 38% of cases, the diagnosis of gastric perforation can only be confirmed postoperatively through intraoperative biopsies [9].

The decision-making process regarding the treatment of PGC involves various factors related to the emergency nature of the condition such as the severity of peritonitis, hemodynamic instability, sepsis, patient comorbidities, and the identification of metastases during exploration. The presence of these variables significantly influences the choice of treatment for PGC, whether it be a preoperative or intraoperative diagnosis, and subsequently guides the management strategy.

Acute management of perforation of gastric cancer

The primary goal of acute management is to stabilize the patient and address the perforation to prevent further complications. The initial approach involves fluid resuscitation with correction of electrolyte abnormalities, broad-spectrum antibiotics to cover potential pathogens involved in intra-abdominal infection, and nasogastric suctioning to drain the stomach [1].

Surgical management of perforation of gastric cancer

Surgical intervention is the mainstay of the treatment, and emergency laparotomy should be performed to repair the perforation and remove the affected portion of the stomach. This may involve either simple closure of the perforation or more extensive procedures, such as partial or total gastrectomy, depending on the extent of the tumor and the overall condition of the patient. In cases where the patient's condition is too unstable for immediate surgery, temporary measures such as peritoneal lavage or placement of a temporary abdominal closure device may be considered to stabilize the patient before definitive surgical intervention [1]. Close postoperative monitoring and appropriate supportive care are crucial to optimize the patient's recovery and minimize postoperative complications. Overall, the acute management of perforated gastric cancer requires a multidisciplinary approach involving surgeons, anesthesiologists, and intensivists to ensure timely and effective treatment for this life-threatening condition.

There are two goals of surgery in patients with PGC: to relieve the peritonitis and to achieve a curative resection. However, the standard surgical protocol remains unclear and largely depends on the individual assessment of the patient. The ultimate decision depends on the patient’s conditions - hemodynamic stability, comorbidities, and the extent of the disease. In the past, primary closure or emergent simple resection was the most common solution due to the poor prognosis of PGC, which was associated with a high degree of postoperative mortality [1]. However, this outcome is likely biased, as typically patients who were frail and with end-stage cancer were chosen as a last-ditch effort [10].

Studies have demonstrated that performing curative-intent resection with gastrectomy yields favorable outcomes in the management of GC [11]. Patients in all stages of GC benefit from a curative-intent, R0 resection, which significantly improves median survival and long-term prognosis [11,12]. Severe reports on PGC indicated that patients undergoing two-stage gastrectomy experienced significantly higher median survival and five-year survival rates compared to those undergoing single-stage gastrectomy [11,12]. The two-stage gastrectomy approach involves initial surgical management of the perforation, followed by curative R0 resection [11-14]. The aim is to control the systemic effects of peritonitis and obtain a biopsy for histological evaluation and accurate staging before planning a radical oncological procedure [11-14].

There is a growing trend towards the use of laparoscopic techniques in the management of PGC. Laparoscopy offers improved visualization of the entire abdominal cavity compared to open laparotomy and reduces postoperative complications, such as adhesions, which can affect subsequent surgeries like two-stage gastrectomy [15,16]. Furthermore, studies have demonstrated comparable overall and disease-free survival rates between laparoscopic and open gastrectomies [15,16]. However, it is important to note that laparoscopic GC surgery requires specialized surgeons, particularly in emergent situations, and is not yet considered the standard of care [16].

Role of chemotherapy in perforation of gastric cancer

The role of chemotherapy in the management of PGC is unclear. In general, the use of chemotherapy in GC is more commonly employed in advanced or metastatic cases, as adjuvant or neoadjuvant therapy to surgery, or in cases where the tumor is not amenable to surgical resection. However, there is limited research on the specific use of chemotherapy in PGC. The decision to use chemotherapy in PGC should be made on a case-by-case basis, taking into consideration factors such as the patient's overall health, tumor characteristics, and individual treatment goals. It is recommended to discuss the potential benefits and risks of chemotherapy with a multidisciplinary team, including oncologists and surgeons, to determine the most appropriate treatment approach for each patient.

Hyperthermic intraperitoneal chemotherapy (HIPEC) has emerged as a potential adjunctive treatment in PGC, aiming to address the high risk of peritoneal metastasis and local recurrence. In HIPEC, chemotherapy agents are administered directly into tumor tissue while heating the abdominal cavity [17,18]. HIPEC has also been reported to have favorable intraoperative blood loss, postoperative outcomes, and shorter hospital stays [19]. Although limited by the lack of well-designed clinical trials, retrospective studies suggest that HIPEC may improve survival rates, reduce peritoneal recurrence, and increase disease-free intervals, particularly in patients with limited peritoneal disease burden and adequately resected primary tumors [20]. However, further research is needed to establish the optimal use of HIPEC in PGC, including refining patient selection criteria, standardizing HIPEC techniques, and investigating the optimal timing and chemotherapy regimens.

## Conclusions

PGC is a devastating complication of advanced GC that requires immediate surgical intervention due to its high mortality rate and systemic complications. The current gold standard treatment for PGC involves a surgical resection with R0 resection, which offers the best long-term outcomes. Additionally, HIPEC shows promising outcomes in improving overall and disease-free survival rates while reducing mortality. However, due to the rarity of PGC as a specific outcome of advanced GC, further research is crucial to establish the optimal standard treatment for this challenging condition. Therefore, continued efforts in research and clinical trials are needed to enhance our understanding and management of PGC, ultimately improving patient outcomes and quality of life.

## References

[1] Bertleff MJ, Lange JF (2010). Perforated peptic ulcer disease: a review of history and treatment. Dig Surg.

[2] Nirula R (2014). Gastroduodenal perforation. Surg Clin North Am.

[3] Bray F, Ferlay J, Soerjomataram I, Siegel RL, Torre LA, Jemal A (2018). Global cancer statistics 2018: GLOBOCAN estimates of incidence and mortality worldwide for 36 cancers in 185 countries. CA Cancer J Clin.

[4] Xia JY, Aadam AA (2022). Advances in screening and detection of gastric cancer. J Surg Oncol.

[5] Hwang SW, Lee DH (2014). Is endoscopic ultrasonography still the modality of choice in preoperative staging of gastric cancer?. World J Gastroenterol.

[6] Pasechnikov V, Chukov S, Fedorov E, Kikuste I, Leja M (2014). Gastric cancer: prevention, screening and early diagnosis. World J Gastroenterol.

[7] Khanderia E, Markar SR, Acharya A, Kim Y, Kim YW, Hanna GB (2016). The Influence of Gastric Cancer Screening on the Stage at Diagnosis and Survival: A Meta-Analysis of Comparative Studies in the Far East. J Clin Gastroenterol.

[8] Lui FH, Tuan B, Swenson SL, Wong RJ (2014). Ethnic disparities in gastric cancer incidence and survival in the USA: an updated analysis of 1992-2009 SEER data. Dig Dis Sci.

[9] Melloni M, Bernardi D, Asti E, Bonavina L (2020). Perforated gastric cancer: a systematic review. J Laparoendosc Adv Surg Tech A.

[10] Tsujimoto H, Hiraki S, Sakamoto N (2010). Outcome after emergency surgery in patients with a free perforation caused by gastric cancer. Exp Ther Med.

[11] Lehnert T, Buhl K, Dueck M, Hinz U, Herfarth C (2000). Two-stage radical gastrectomy for perforated gastric cancer. Eur J Surg Oncol.

[12] Fisher BW, Fluck M, Young K, Shabahang M, Blansfield J, Arora TK (2020). Urgent surgery for gastric adenocarcinoma: a study of the National Cancer Database. J Surg Res.

[13] Hata T, Sakata N, Kudoh K, Shibata C, Unno M (2014). The best surgical approach for perforated gastric cancer: one-stage vs. two-stage gastrectomy. Gastric Cancer.

[14] Mahar AL, Brar SS, Coburn NG, Law C, Helyer LK (2012). Surgical management of gastric perforation in the setting of gastric cancer. Gastric Cancer.

[15] Bertleff MJ, Halm JA, Bemelman WA (2009). Randomized clinical trial of laparoscopic versus open repair of the perforated peptic ulcer: the LAMA Trial. World J Surg.

[16] Quan Y, Huang A, Ye M (2016). Comparison of laparoscopic versus open gastrectomy for advanced gastric cancer: an updated meta-analysis. Gastric Cancer.

[17] Tsuyoshi H, Inoue D, Kurokawa T, Yoshida Y (2020). Hyperthermic intraperitoneal chemotherapy (HIPEC) for gynecological cancer. J Obstet Gynaecol Res.

[18] Revaux A, Carbonnel M, Kanso F, Naoura I, Asmar J, Kadhel P, Ayoubi JM (2020). Hyperthermic intraperitoneal chemotherapy in ovarian cancer: an update. Horm Mol Biol Clin Investig.

[19] Fagotti A, Petrillo M, Costantini B (2014). Minimally invasive secondary cytoreduction plus HIPEC for recurrent ovarian cancer: a case series. Gynecol Oncol.

[20] Khan H, Johnston FM (2022). Current role for cytoreduction and HIPEC for gastric cancer with peritoneal disease. J Surg Oncol.

